# Resilience to social stress: is it in the blood?

**DOI:** 10.1002/2211-5463.13291

**Published:** 2021-10-02

**Authors:** Ruzhica Bogeska

**Affiliations:** ^1^ FEBS Open Bio Editorial Office Cambridge UK

## Abstract

In social mammalian species, social stress can arise from different social interactions. Repeated exposure to social stressors can lead to neuropathology and psychiatric disorders. In this issue, Sakamoto *et al*. report on alterations in extracellular vesicles (EVs) in a mouse model of chronic social defeat stress (CSDS). The data suggest that mice susceptible to CSDS have alterations in the miRNA content of circulating EVs, which influences the expression of pro‐inflammatory cytokines in microglia cells.

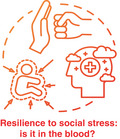

**Abbreviations**
CSDS: chronic social defeat stress
EV: extracellular vesicle

In social mammalian species, such as human and mouse, social stress can arise from different social interactions, for example, from encounters with aggressive strangers (whether they be mice or men). Repeated exposure to such stress can lead to various conditions, including depression and anxiety, but different individuals show varying degrees of resilience to such stress. To explore the mechanisms underlying such differential response to stress, Sakamoto *et al*. from Johns Hopkins University and University of Alabama at Birmingham used a mouse model of chronic ‘social defeat’ stress (CSDS) to examine molecular changes in the blood following stress. In an article published in this issue of *FEBS Open Bio*, the authors report how molecular alterations in circulating extracellular vesicles (EVs) influence social behaviours and resilience to chronic social stress. Test mice were classified as resilient or susceptible to social stress, and their blood samples were analysed. Their data demonstrated that mice susceptible to CSDS have alterations in the miRNA content of circulating EVs, and these EVs in turn influence the expression of pro‐inflammatory cytokines in microglia cells, thereby resulting in social avoidance phenotypes. Since low resilience to social stress may be linked to the progression of several psychiatric disorders, it may ultimately be possible to determine prognosis or likely response to therapy for individuals susceptible to or suffering from such conditions by profiling pro‐inflammatory cytokines and EV‐associated miRNAs in blood samples.

Research on the effect of social stress on physiological functions gained attention as early as the beginning of the 20th century, and it has been reported in the works of Walter Cannon [1] and Hans Selye [2]. Phrases like ‘Emotional derangement in bodily functions’ or ‘the stress of life’ outline the sentiment of this early work.

What is social stress? What are the behavioural and physiological consequences of repeated exposure to social stress, and does each individual respond in the same way? How does the body react and how can these processes be studied? These are a few of the questions that have sparked the interest of researchers from various fields, including those with a cellular and molecular biology background.

In social mammalian species, such as human and mouse, social stress can arise from different social interactions, for example, from encounters with aggressive strangers. Repeated exposure to social stressors can in turn lead to abnormal behaviours, neuropathology and even psychiatric disorders, such as anxiety and depression [3, 4]. Various rodent models have been employed to study the behavioural and physiological changes during social stress and social defeat [5], such as the mouse model of chronic social defeat stress (CSDS). In this paradigm, an alpha resident male mouse (resident aggressor) is first introduced to an intruder mouse, and this is followed by examining the mouse's response to the so‐called ‘three chamber social interaction test’, which allows researchers to determine the behavioural and physiological changes following CSDS. This test is illustrated in Fig. 1.

**Fig. 1:**
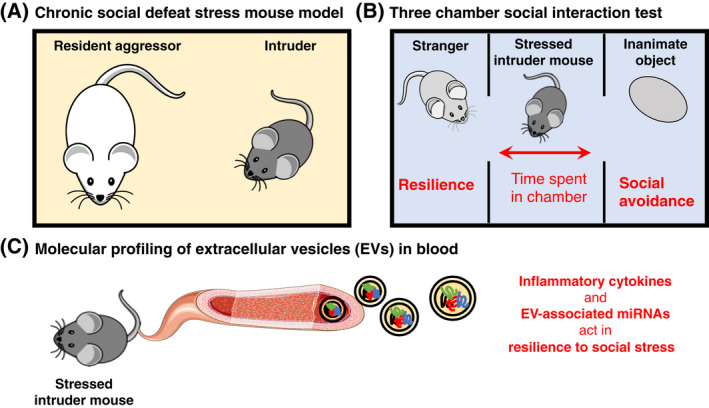
Schematic representation of the workflow used by Sakamoto *et al*. (A) The authors used a mouse model of CSDS in which an intruder mouse was introduced to the housing cage of an alpha resident male mouse (resident aggressor) for 10 days. (B) On day 11, the social behaviour of the intruder mouse exposed to CSDS was assessed using the ‘three chamber social interaction test’ that includes a housing cage separated into three compartments. The test mouse is placed in the middle compartment and given the option to interact with either a nonaggressive stranger mouse in one adjoining compartment, or with an inanimate object similar in size and shape to the stranger mouse in the other adjoining compartment. The researchers can then estimate resilience to social stress based on the amount of time the mouse spends in each of the chambers. (C) Finally, the researchers examined circulating inflammatory cytokines and EVs in the blood of the test mouse on day 12.

With the advance of modern technologies and methods in biology over the past few decades, it has become possible to investigate the cellular and molecular processes underlying social stress behaviours in much greater detail. For instance, intracerebral changes in immune cells (also termed as microglia), as well as changes in inflammatory cytokines and chemokines in cerebrospinal fluid, plasma and blood, have been found to be related to exposure to chronic social stress in patients with stress‐related psychiatric disorders such as depression [6], as well as in CSDS mouse models [7, 8].

While the link between inflammation and mental disorders is now well established, the origin and cause of perturbed inflammatory signalling during social stress and mental illness remains to be discovered. Recently, extracellular vesicles (EV) have gained attention in the context of neuroinflammation and mental disorders [9]. EVs are defined as small lipid bilayer particles that originate from various cell types and carry molecular cargo throughout the circulation to distant cells [10]. It has been suggested that EVs circulating in bodily fluids may be of use for the diagnosis and treatment of mental disorders [11], and they may also be linked to the physiological response to mental and social stress.

In this issue of *FEBS Open Bio*, Dr. Shin‐ichi Kano, Dr. Atsushi Kamiya and colleagues report on the molecular alterations in EVs which underlie social stress‐induced behaviours in mice [12]. The authors used a previously established CSDS mouse model in which individual test mice were introduced to a resident aggressor mouse for 10 days in a row (Fig. 1A). On the day after this process, the social stress response of the test mice was estimated using the ‘three chamber social interaction test’ and the test mice were classified as resilient or susceptible to social stress (Fig. 1B). The authors found that social avoidance phenotypes strongly correlated with higher expression of pro‐inflammatory cytokines, in line with previous observations.

Since miRNAs derived from blood EVs have been previously linked to major depressive disorder in patients (13), Dr. Kano and colleagues decided to examine whether the miRNA content in EVs from CSDS mice was related to social avoidance phenotypes. Indeed, the authors observed that a subset of EV‐associated miRNAs involved in regulating the expression of pro‐inflammatory cytokines are predictive of social avoidance phenotypes (Fig. 1C). Additionally, experiments with a microglia‐like cell line showed that cells exposed to EVs from mice susceptible to social stress exhibit increased expression of pro‐inflammatory cytokines. Altogether, the data suggest that mice susceptible to CSDS have alterations in the miRNA content of circulating EVs, and these EVs in turn influence the expression of pro‐inflammatory cytokines in microglia cells, thereby resulting in social avoidance phenotypes.

Although the study investigated a small mouse cohort and more work is required to explore any potential translation to human studies, the research highlights the importance of EV‐associated miRNAs and inflammation in physiological resilience to social stress. Since low resilience to social stress may be linked to the progression of several psychiatric disorders, it may ultimately be possible to determine prognosis or likely response to therapy for individuals susceptible to or suffering from such conditions by profiling pro‐inflammatory cytokines and EV‐associated miRNAs in blood samples.

## Conflict of interest

The author declares no conflict of interest.

## Author contribution

RB wrote the article and prepared the figure.
